# The Transcriptional Response to Nonself in the Fungus *Podospora anserina*

**DOI:** 10.1534/g3.113.006262

**Published:** 2013-06-01

**Authors:** Frédérique Bidard, Corinne Clavé, Sven J. Saupe

**Affiliations:** * Université de Paris Sud, Institut de Génétique et Microbiologie, UMR8621 Orsay, France, and; † IBGC UMR 5095 CNRS Université Bordeaux Segalen, Bordeaux, France

**Keywords:** *Podospora anserina*, heterokaryon incompatibility, nonself recognition, STAND proteins, HET domain

## Abstract

In fungi, heterokaryon incompatibility is a nonself recognition process occurring when filaments of different isolates of the same species fuse. Compatibility is controlled by so-called *het* loci and fusion of strains of unlike *het* genotype triggers a complex incompatibility reaction that leads to the death of the fusion cell. Herein, we analyze the transcriptional changes during the incompatibility reaction in *Podospora anserina*. The incompatibility response was found to be associated with a massive transcriptional reprogramming: 2231 genes were up-regulated by a factor 2 or more during incompatibility. In turn, 2441 genes were down-regulated. HET, NACHT, and HeLo domains previously found to be involved in the control of heterokaryon incompatibility were enriched in the up-regulated gene set. In addition, incompatibility was characterized by an up-regulation of proteolytic and other hydrolytic activities, of secondary metabolism clusters and toxins and effector-like proteins. The up-regulated set was found to be enriched for proteins lacking orthologs in other species and chromosomal distribution of the up-regulated genes was uneven with up-regulated genes residing preferentially in genomic islands and on chromosomes IV and V. There was a significant overlap between regulated genes during incompatibility in *P. anserina* and *Neurospora crassa*, indicating similarities in the incompatibility responses in these two species. Globally, this study illustrates that the expression changes occurring during cell fusion incompatibility in *P. anserina* are in several aspects reminiscent of those described in host-pathogen or symbiotic interactions in other fungal species.

Fungi generally inhabit crowded ecological niches and are therefore engaged in a variety of biotic interactions with other eukaryotic and bacterial species and with other individuals belonging to the same species (Druzhinina *et al.* 2011; Frey-Klett *et al.* 2011; Leeder *et al.* 2011; Veneault-Fourrey and Martin 2011). During such interactions, ranging from mutualism to pathogenesis, recognition and appropriate response to nonself is essential. Comparative genomics suggest that fungi dedicate a large part of their genomes to such niche adaptation processes and that functional categories such as small secreted cysteine-rich proteins, secondary metabolism clusters, proteases and lipases, and carbohydrate-active enzymes are among the key players in such biotic interactions (Martin *et al.* 2008; Spanu and Kamper 2010; Ohm *et al.* 2012). The genes involved in these processes, in particular pathogenicity, tend to be enriched in specific, variable regions of the genome or even on supernumerary dispensable chromosomes (Rep and Kistler 2010; Schmidt and Panstruga 2011).

Recognition and response to conspecific nonself in filamentous fungi takes the form of heterokaryon incompatibility, a nonself rejection process that takes place when filaments of unlike compatibility type undergo cell fusion (Saupe 2000; Glass and Dementhon 2006; Aanen *et al.* 2010). Compatibility is determined by so-called *het* loci (for heterokaryon incompatibility). Any given species displays generally half a dozen to a dozen of such loci that act redundantly so that a genetic difference for a single *het* locus is sufficient to trigger the incompatibility reaction. *het*-loci generally display high levels of polymorphism. The severity of the incompatibility reaction can vary depending on the *het* locus involved but the reaction generally leads to the lytic cell death of the heterokaryotic fusion cells. It is proposed that the biological role of incompatibility is protection against the spreading of deleterious cytoplasmic elements such as senescence plasmids and mycoviruses or protection against conspecific parasitism (Caten 1972; Hartl *et al.* 1975; Debets *et al.* 1994; Hoekstra 1994; Aanen *et al.* 2008). The “Crozier-paradox” predicts, however, that allorecognition systems are evolutionary unstable and that external selective forces may be required for maintenance of polymorphism at allorecognition loci (Crozier 1986; Rousset and Roze 2007; Aanen *et al.* 2008). We have proposed that the evolutionary origin of the incompatibility response could reside in a more general function of response to heterospecific nonself (xenorecognition) (Paoletti and Saupe 2009). Specifically, we have hypothesized, as already proposed in the case of plant hybrid necrosis or graft rejection in mammals, that incompatibility could result from pathogen-driven divergence in host defense genes.

Together with *Neurospora crassa*, *Podospora anserina* is one of the main models for the study of incompatibility (Glass and Kaneko 2003; Pinan-Lucarre *et al.* 2007). Nine *het* loci have been described genetically in Podospora and among them five (*het-c*, *het-d*, *het-e*, *het-r*, and *het-s*) have currently been identified. *het-d*, *het-e*, and *het-r* are paralogs belonging to the same gene family termed *hnwd* (Saupe *et al.* 1995; Espagne *et al.* 2002; Paoletti *et al.* 2007; Chevanne *et al.* 2009; Saupe 2011). These genes encode STAND proteins comprising a C-terminal WD-repeat domain, a central NACHT nucleotide binding oligomerization domain, and an N-terminal HET domain (Leipe *et al.* 2004). The WD-repeat domain of these proteins is the nonself recognition domain and was found to be highly variable and subject to positive diversifying selection and concerted evolution between members of the gene family (Espagne *et al.* 2002; Paoletti *et al.* 2007). The HET domain is a proposed cell death execution domain found in many proteins encoded by fungal incompatibility genes (Smith *et al.* 2000; Paoletti and Clave 2007). An exception is the *het-s/het-S* incompatibility system which does not involve a HET domain as cell death execution module. Instead, in this system, toxicity is mediated by a domain termed HeLo, which exerts its toxicity at the cell membrane as a pore-forming toxin (Greenwald *et al.* 2010; Mathur *et al.* 2012; Seuring *et al.* 2012). The domain is also found in the LopB pathogenicity protein of *Leptosphaeria maculans* (Idnurm and Howlett 2003). STAND proteins with NACHT domains appear of general importance in the control of incompatibility because the *het-s/het-S* system also involves a STAND protein termed NWD2 (Daskalov *et al.* 2012). In addition, *vic2* incompatibility in *Cryphonectria parasitica* is also controlled by a STAND protein of the NB-ARC-TPR type (Choi *et al.* 2012).

In Podospora, the incompatibility reaction has been mostly studied using a particular experimental system, the *het-R het-V* self-incompatible (SI) strain (Pinan-Lucarre *et al.* 2007). Most of the knowledge gained on the incompatibility reaction in Podospora has been acquired in this experimental system. The *het-R* and *het-V* loci are unlinked and define a nonallelic incompatibility system, so that the incompatible alleles can be reunited in the same haploid nucleus with an appropriate cross. Conveniently, the *het-R het-V* SI strain is thermosensitive. *het-R/het-V* incompatibility is suppressed at 32° and is triggered by a transfer at 26°, the normal growth temperature of *P. anserina*. The death reaction occurs asynchronously in nearly all cells. A morphological change of the vacuolar compartment from a preexisting tubular network to round vacuoles occurs early on. Large vacuoles occupy most of the cell volume, fuse and finally cell lysis occurs. Cell death appears to result either from vacuolar or cytoplasmic membrane rupture (Pinan-Lucarre *et al.* 2005). The incompatibility reaction is also associated with increased septation, accumulation of lipid droplets, and abnormal deposition of cell wall material. Early biochemical studies have revealed that *het-R/het-V* incompatibility is associated with a global decrease in normal protein translation and transcription while concomitantly novel enzymatic activities such as phenoloxidases, dehydrogenases and proteases appear (Bégueret and Bernet 1973; Boucherie and Bernet 1978; Bourges *et al.* 1998). The incompatibility reaction is associated with regulation of gene expression occurring at least in part at the transcriptional level and leading to the induction of a specific set of genes originally termed *idi* genes (induced during incompatibility) (Bourges *et al.* 1998). Six of them have been characterized. The *idi-1*, *idi-2*, and *idi-3* genes encode small putative secreted proteins. IDI-2 is related to a fungal toxin (de Sa *et al.* 2010a). The *idi-4* gene encodes a bZIP transcription factor that induces expression of other *idi* genes and is able to produce a phenocopy of the incompatibility cell death reaction when over-expressed (Dementhon *et al.* 2004; Dementhon and Saupe 2005). A vacuolar protease accumulating during cell death by incompatibility has been purified (Paoletti *et al.* 2001). The corresponding *pspA* gene is also an *idi* gene (*idi-6/pspA*). Both *idi-6* and *idi-7/PaATG8* genes are involved in autophagy. Autophagy is strongly induced during the incompatibility reaction but the autophagy process is not responsible for cell death since inactivation of the autophagy pathway accelerates rather than suppresses cell death (Pinan-Lucarre *et al.* 2003; Pinan-Lucarre *et al.* 2005).

Transcriptional profiling of the incompatibility reaction has been analyzed previously in *Neurospora crassa* (Hutchison *et al.* 2009). A genome-wide analysis was performed in the context of *het-c/pin-c* incompatible heterokaryon interaction that also involves a HET domain protein and uses a thermosensitive allele of *pin-c*. Incompatibility was triggered by transferring the heterokaryotic strain from 34° to 20°. Transcriptional changes were extensive with more than 3000 genes showing a differential expression, with roughly similar numbers of these being up-regulated and down-regulated genes. Gene expression changes were also analyzed by subtractive hybridization in the filamentous basidiomycete *Amylostereum areolatum* (van der Nest *et al.* 2011). To gain a global picture of the expression changes occurring during incompatibility in *Podospora*, we performed a genome wide transcriptional profiling in the *het-R het-V* SI strain upon the induction of the incompatibility reaction.

## Materials and Methods

### Strains and growth media

The *het-r het-V mat+* strain corresponds to the s wild-type isolate from the Rizet and Bernet collection. The SI *het-R het-V mat+* strain is isogenic to the s strain except for the *het-r* locus. The *het-R* allele was present in the M wild-type isolate from Rizet and Bernet collection. The microarrays used in this study contain 10,556 oligonucleotidic probes whose sequence was derived from the genomic sequenced *S* strain (Espagne *et al.* 2008). The two *s* and *S* wild-type isolates are nearly isogenic except at the *het-s* incompatibility locus as verified through genomic hybridization (Bidard *et al.* 2010). For total RNA preparation, mycelia were grown on solid synthetic M2 medium (see http://podospora.igmors.u-psud.fr./methods.php) as for other Podospora transcriptional analyses (Bidard *et al.* 2011; Bidard *et al.* 2012).

### RNA preparation

The s and SI strains, in collection at −80°, were thawed on solid M2 medium at 32°. The SI phenotype was controlled through the complete absence of growth after 48-hr transfer to 26°. Petri dishes containing M2 medium were covered with a cellophane disc (cat. no. 1650193; BioRad Laboratories, Hercules, CA), prewarmed at 32°, and inoculated with nine explants from *P. anserina* control or SI cultures grown on M2 medium at 32°. The cultures were placed at 32° for 44-hr growth. After 40 hr of growth the cultures were transferred for 4 additional hr onto fresh 32°-prewarmed medium to avoid any effect of starvation on gene expression. After 44 hr of growth at 32°, the nine colonies are confluent on M2 medium. Mycelia were either harvested by scraping with a microscope coverglass (time 0, T0) or transferred onto fresh 26°-prewarmed M2 medium before further incubation at 26°. After different times at 26° (30 min, 1 hr, 2 hr, 3 hr, or 4 hr) the cultures were harvested. The mycelium was then stored in aliquots of no more than 100 mg in liquid nitrogen until RNA extraction. Each plate was considered to be a biological replicate. Four biological replicates were prepared for each time point. RNA extractions were performed as described (Bidard *et al.* 2011). The quantity and quality of the total RNA were determined using a Nanodrop spectrophotometer (Nanodrop technologies, Wilmington, DE) and the Bioanalyzer 2100 system (Agilent, Santa Clara, CA) as described previously (Imbeaud *et al.* 2005).

### Labeling of cDNA and microarray hybridization

Gene expression microarrays for *P. anserina* consisted of a 4 × 44 K platform (AMADID 018343, Agilent, Santa Clara, CA) containing 10,556 probes on each array with each probe present in four replicates (Bidard *et al.* 2011). Microarray hybridization experiments, including target preparation, hybridization, and washing, were performed as described (Bidard *et al.* 2011). The labeling efficiency and the product integrity were checked as described previously (Imbeaud *et al.* 2005). Four biological replicates labeled with Cy-3 for each of the different experimental condition were compared with a common reference labeled with Cy-5, in indirect comparisons. The common reference was obtained by mixing RNA extracted from different growth conditions as described (Bidard *et al.* 2010). A total of 825 ng of each of the Cy3- and Cy5-labeled targets was mixed and incubated on microarray slides for hybridization.

### Microarray data acquisition and analyses

Microarrays were scanned with the Agilent DNA microarray Scanner (Agilent) at a resolution of 5 microns/pixel using the extended dynamic range function. Spot and background intensities were extracted with the Feature Extraction (v9.5.3) software (Agilent) using the GE2-v4_95_Feb07 default protocol. Array quality was assessed through Agilent control features as well as spike-in controls (Agilent 2-Color Spike-in Kit for RNA experiment). Subsequent flagging was done according to the GenePix Pro software (Molecular Devices Sunnyvale, CA) nomenclature, which included four flag levels (good [100], bad [−100], not found [−50], moderate [0]). Preprocessing and data normalization were performed with Feature Extraction software (Agilent technologies), with the GE2-v4_95_Feb07 default protocol. Statistical differential analysis were done using the MAnGO software (Marisa *et al.* 2007), a moderate *t*-test with adjustment of *P*-values (Benjamini 1991) was computed to measure the significance of each expression difference. Differential analyses have been performed between all the time points at 26° and time T0 for each strain. Fold change values (FC) were calculated from the ratio (R) between normalized intensities at each time in the comparison. FC = R when R > 1; FC = −1/R when R < 1. Genes were considered as differentially expressed during incompatibility if their absolute fold change of up or down regulation in the SI strain was greater than 2 with an adjusted *P*-value <0.001 and if no differential expression was detected in wild-type control.

### Data clustering

A total of 2285 coding sequences (CDS) that were differentially expressed in the SI strain between 32° and 26° (-fold change >4 with *P* < 0.001) were grouped. A first hierarchical clustering (unweighted pair group method with arithmetic mean, *i.e.*, UPGMA, method) of this set of genes defined two classes: genes up-regulated (1036 CDS) and genes down-regulated (1249 CDS). A hierarchical clustering (UPGMA method) and a K-mean clustering to 12 classes were computed independently using Spotfire Decision Site software package (Spotfire, Somerville, MA) with correlation coefficient as similarity measure on the 1036 up-regulated genes. Intersection of the clusters obtained by the two clustering methods resulted in eight robust clusters. A total of 999 CDS have been classified and 37 CDS were not robustly classified.

### Functional annotation

For Gene Ontology (GO) term enrichment analyses, the CDS have been annotated with BLAST2GO (http://www.blast2go.org/) and subsequently analyzed with cateGOrizer (http://www.animalgenome.org/bioinfo/tools/catego/) using the GO_slim classification setting. Pfam domain enrichment analyzes were carried out by performing a batch annotation of the *P. anserina* proteins at http://pfam.sanger.ac.uk/. The list of predicted extracellular proteins was retrieved at http://proteomics.ysu.edu/secretomes/fungi.php (Lum and Min 2011). All enrichment *P*-values were calculated with a two-tailed Fisher-test. Secondary metabolism clusters were identified and analyzed with antismash at http://antismash.secondarymetabolites.org/ (Medema *et al.* 2011). *P. anserina* and *N. crassa* orthologous gene pairs were identified using FUNGIpath (Grossetete *et al.* 2010). Transposon sequence counting was restricted to the most frequent elements, the *crapaud* and *discoglosse* elements (Espagne *et al.* 2008). The list of genes encoding carbohydrate active enzymes was recovered at CAZy (http://www.cazy.org/).

### Analyses of chromosome-specific enrichments of coregulated genes and of occurrence of clusters of adjacent regulated genes

We calculated the *P*-value for chromosome-specific enrichment of up-regulated genes with a two-tailed Fisher-test by comparing the observed occurrence of up-regulated genes with the expected random distribution. For the calculation of the expected number of clusters of adjacent coregulated genes occurring in a random distribution, we consider the genome as single concatenated molecule. The value of *c*, the expected number of clusters of adjacent coregulated genes of a given size, occurring in a random distribution, was calculated as *c*_n_= *f*^n^.g, where g is the total number of genes (10,556) and *f* is the fraction of up- or down-regulated genes (respectively, 2231/10,556 and 2441/10,556). Therefore, for instance, in a random distribution the number of expected cluster of three adjacent up-regulated genes would be *c*_3_= (2231/10,556)^3^. 10,556. Values of *c* were calculated for n = 2−10. Then, to obtain the number of clusters of a given size, we corrected for the occurrence of clusters embedded in clusters of a larger size. The number of clusters of exactly nine adjacent genes expected at random was N_9_= *c*_9_− 2N_10_ because a cluster of 10 adjacent coregulated genes contains two clusters of nine adjacent coregulated genes (N_10_ was equated to *c*_10_). Similarly, N_8_ the number of clusters of exactly 8 adjacent genes as *c8-2*N*_9_*-*3*N*_10_*, N*_7_* the number of clusters of exactly seven adjacent genes as c_7_-2N_8_-3N_9_-4N_10_ and so on. To empirically validate this approach, we ran a numerical simulation by generating 20 sets of 2231 numbers (the number of up-regulated genes) in a range of 1 to 10,556 (the total number of genes) at www.randomizer.org/form.html. In each set the number of occurrences of series of contiguous numbers of a given size (for 2−10) was obtained and averaged over the 20 sets. The results of the numerical simulation were identical to the values calculated as described above within ±10%.

### Microarray data accession number

All microarray data are MIAME compliant. The raw data has been deposited in the MIAME compliant Gene Expression Omnibus database (Edgar *et al.* 2002) and is accessible through the GEO Series accession number GSE21659 (http://www.ncbi.nlm.nih.gov/geo/query/acc.cgi?acc=GSE21659).

## Results

### Extensive transcriptional reprogramming during incompatibility

**Figure 1:**
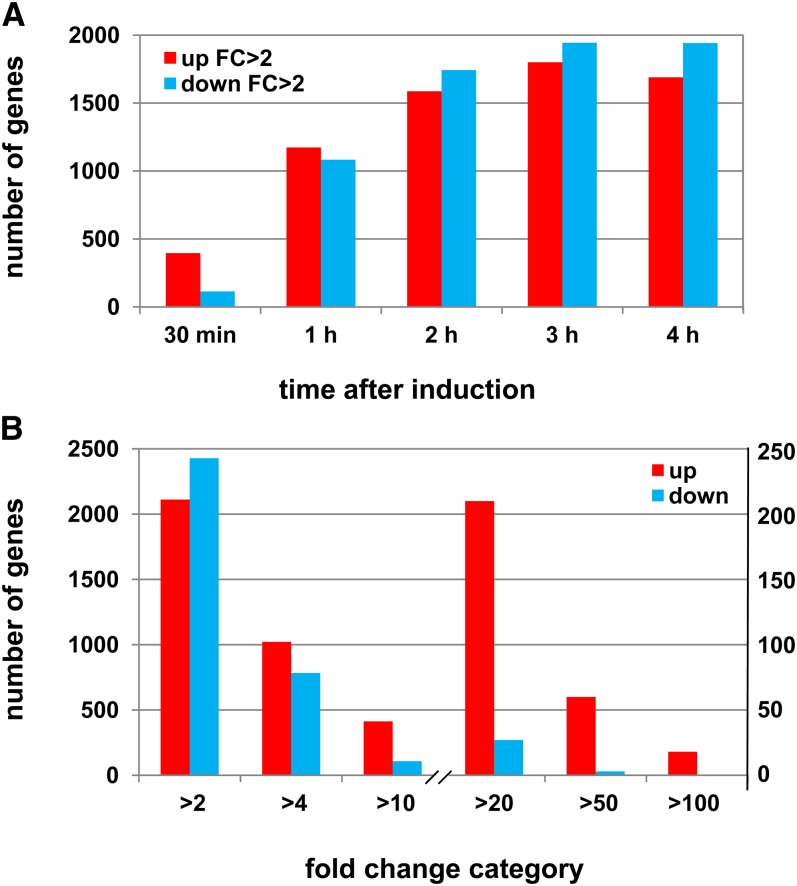
Global kinetics and magnitude of gene expression changes during incompatibility. In (A), the number of up- and down-regulated genes at each time point is given. Note that a comparable number of genes constitute the up- and down-regulated categories except at 30 min. In (B), the number of up- and down-regulated genes in each maximum fold change category is given. The scale on the left and right are for the first and last three categories respectively. Note that the up-regulated gene set contains more genes with elevated fold change values.

To analyze gene expression changes during incompatibility, we used an experimental system in which the incompatibility reaction is conditional and can be strictly controlled, namely the *het-R het-V* SI strain (Labarere 1973). *het-R/het-V* incompatibility is conditional; at 32° this strain grows as wild type but the transfer to 26°, triggers the incompatibility reaction: the growth stops, the cytoplasm shows extensive vacuolization, hyphal compartmentation increases, autophagy is induced, and ultimately cells gradually die (Pinan-Lucarre *et al.* 2005). This SI strain and the control self-compatible *s* strain were grown for 44 hr at 32° and then transferred to 26°. RNA was extracted from the two strains before transfer and at different times after transfer to 26° and prepared for hybridization to gene expression microarrays developed for *P. anserina* (Bidard *et al.* 2010). We compared the transcriptional profiles of the *het-R het-V* and control *s* strains after growth at 32° and after transfer to 26° for 30 min, 1 hr, 2 hr, 3 hr, and 4 hr. A set of 2231 genes were found to be up-regulated (Supporting Information, Figure S1). This group was composed of 2115 genes up-regulated by a fold change of two or more and also comprises 116 genes whose expression is only detected during the incompatibility reaction. This group of genes was designated incompatibility-specific (*isp*). The down-regulated gene set comprised 2441 genes down regulated by a fold change of two or more. Figure 1A presents the time course of induction and repression of these two gene sets. Gene up-regulation starts as early as 30 min after transfer to 26° with already induction of roughly 500 genes. The number of up-regulated genes increased strongly during the first 2 hr and then only slightly between 2 and 4 hr after transfer. The same was true for the time course of gene down-regulation. The number of down-regulated genes at 30 min, however, was much lower than in the up-regulated set. In Figure 1B, we plotted the number of genes by maximal fold change in both the up and down set. The fold change range is wide for the up-regulated gene set with a significant proportion of the population displaying elevated fold changes. A total of 433 genes have a FC > 10 and 63 genes have a FC > 50. The same categories only comprise 143 and 5 genes, respectively, in the down-regulated set. Three genes were totally silenced during incompatibility (Table S1).

This transcriptional profiling indicates that during the course of the incompatibility reaction, 44% of the *P. anserina* genes experience a change in their mRNA abundance by a FC of 2 or more, indicating that the incompatibility response induces a major reorganization of the transcriptome.

### Expression profile clusters

**Figure 2:**
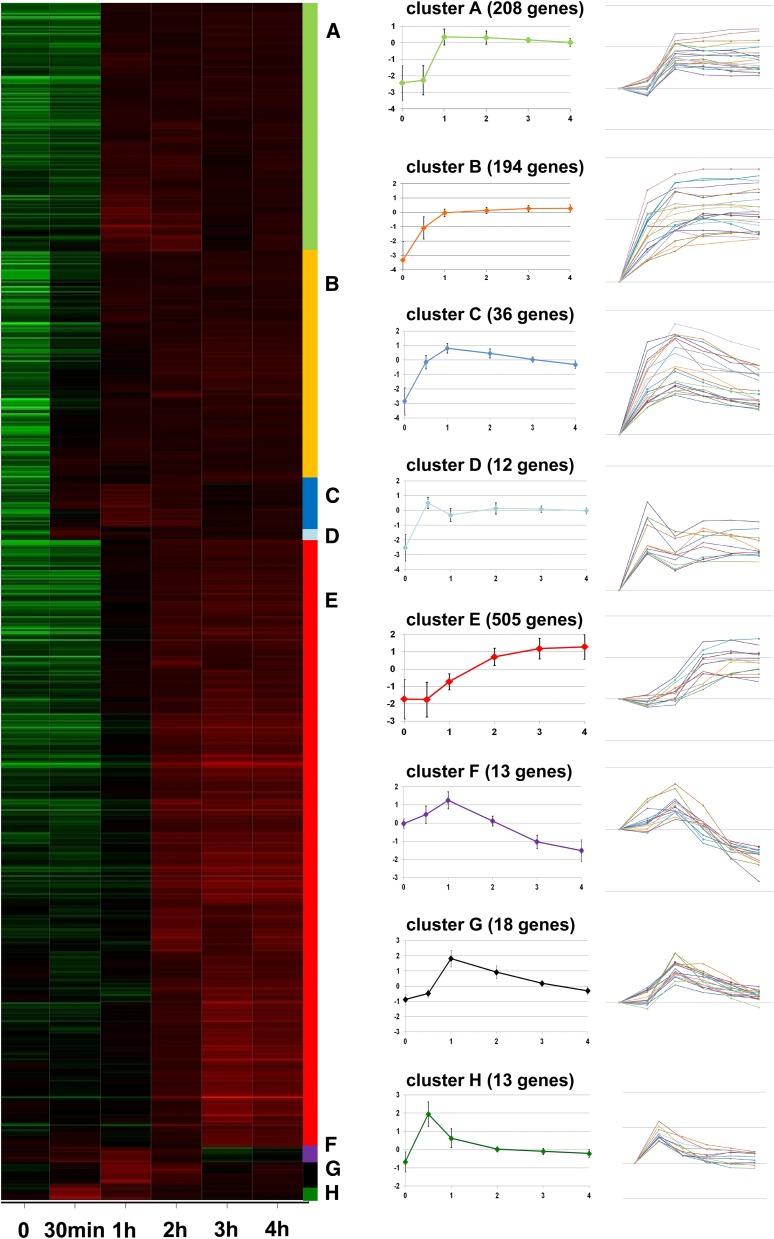
Clustering of up-regulated genes according to their expression profile. On the left the time course expression patterns of 999 up-regulated genes leading to the definition of the eight clusters (A−H) are given. Green and red indicate expression levels above and below the median level, respectively, at each time point after transfer at 26°. For each cluster the average expression profile is given, the 0 value corresponds to the median expression level for each given cluster, error bars are SD values. On the right, the individual fold change values of 15 to 20 genes chosen at random in each cluster are given. For poorly populated clusters, all genes of the cluster are represented. A log scale is used to facilitate comparison of genes with disparate fold change values.

Using the fold-change values at the different time points, we performed clustering of the genes by expression profile on a subset of regulated genes (*i.e.*, those with maximal fold change >4). The hierarchical clustering on the up-regulated gene set led to the identification of 8 robust clusters (clusters A to H; Figure 2). The clusters were variable in size, ranging from 12 to 505 genes. Clusters D and H contain genes that show an early induction. In cluster H, expression peaks already at 30 min then decreases to the initial level whereas in cluster D, expression also peaks early but does not decrease to the initial level. In all other clusters except E, expression peaks roughly at 1 hr and is stably maintained (A and B) or decreased either slightly (C) or to the initial level (G) during the course of the incompatibility reaction. The same is true in cluster F but in this case the genes are down-regulated at later time points. Genes in clusters A and B differ in that cluster A shows a lag phase in the induction. Genes in cluster E are induced late and their expression reaches a plateau after 3h. The clusters with expression peaks (D, F, G, and H) are the least populated while the clusters that show a steady increase or a plateau phase (A, B, and E) contain the large majority of the clustered induced genes. Globally, a large fraction of the genes continue to increase in expression throughout the incompatibility reaction, 893 genes show their maximum fold change at 4 hr.

As mentioned previously, genes in cluster H show a transient early induction peak. It is tempting to speculate that upstream regulators of gene induction during incompatibility might be found in this cluster. Of note is that this cluster of 13 genes is apparently enriched in transcription regulators as it contains 4 genes encoding transcription factors (Pa_1_18880, Pa_3_1720, Pa_1_16130, Pa_2_6830), the first two of which are also up-regulated during incompatibility in *N. crassa* (see section comparison with *N. crassa*). These genes might represent prime candidates for the identification of early and upstream regulators of the incompatibility response. No robust clustering was obtained for the down-regulated gene set.

### Functional annotation of up-regulated and down-regulated genes

**Figure 3:**
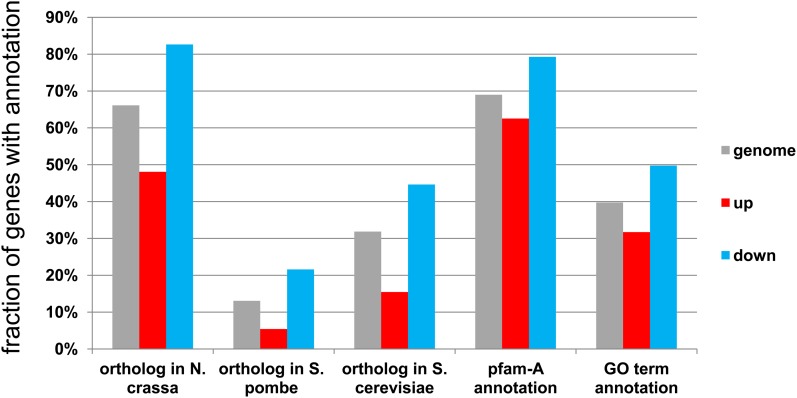
Up- and down-regulated genes are enriched respectively in nonannotated and annotated genes. Various annotations types of up or down gene sets are compared to whole genome annotation. All values were analyzed by statistical Fisher test to evaluate the significance of the difference with respect to the whole genome annotation, all *P* <10^−10^.

Functional annotation of the up- and down-regulated gene sets revealed an overrepresentation in the up-regulated gene set of orphan and nonannotated genes compared with the rest of the genome and, conversely, an enrichment for genes presenting a functional annotation and orthologs in other fungal species in the down regulated gene set (Figure 3). For instance, the proportion of genes showing a *N. crassa* ortholog was 48% in the up-regulated gene set and 82% in the down-regulated gene set. The proportion of genes showing orthologs in *Saccharomyces cerevisiae*, were 13.5% and 41.5%, respectively, in the up- and down-regulated sets. The same bias was observed when considering the fraction of genes displaying a Pfam-A annotation or a gene ontology annotation (GO term). Again, genes with a functional annotation tag of that type were less abundant in the up-regulated gene set and enriched in the down-regulated gene set. The low level of functional annotation was particularly striking in the set of 116 incompatibility specific genes (*isp*). Only 14 (12%) had an ortholog in *N. crassa* (*P* < 10^−30^), and none had orthologs in *S. cerevisiae* or *Schizosaccharomyces pombe*; only 40.5% were annotated with pfam-A domains (*P* < 10^−9^). On the basis of these observations, genes of the down-regulated set appear to belong preferentially to the conserved fungal genome core whereas the up-regulated set appears globally more rapidly divergent and species specific.

To classify the genes into functional categories, we analyzed the GO term enrichment in the up-regulated and down-regulated gene sets (Table 1). In the up-regulated gene set there was significant GO term enrichment for “proteolysis and peptidolysis,” “effectors and modulators, cell communication” (which mainly corresponds to the children terms “signal transduction” and “response to external stimuli”), “protein modification,” and “lipid metabolism.” In the down-regulated gene set the enriched terms were “energy/TCA cycle” (corresponding to children terms “cellular respiration, electron transport chain”), “protein synthesis,” “DNA synthesis and replication,” and “ribosomal proteins.” In general, a GO term enriched in the up-regulated gene set was found to be underrepresented in the down-regulated gene set and *vice versa* (Table 1). The GO term annotation evokes a general shut down of the basic cell processes such as energy production, ribosome function, and DNA replication and an activation of proteolysis, protein modification, and signal transduction processes.

**Table 1:** GO term enrichment in up- and down-regulated gene sets

GO term	Description	GO Term Enrichment in Up-Regulated Gene Set	*P*-Value of Enrichment	GO Term Enrichment in Down-Regulated Gene Set	*P*-Value for Enrichment
GO:0006508	Proteolysis and peptidolysis	**2.2**	4.00E-05	0.36	4.50E-07
GO:0007154	Effectors/modulators; cell communication	**1.88**	4.00E-04	0.42	1.00E-06
GO:0006464	Protein modification	**1.64**	1.20E-02	0.33	9.00E-09
GO:0006629	Lipid metabolism	**1.59**	3.20E-02	1.15	n.s.
GO:0006091	Energy/TCA cycle	0.27	1.00E-02	**1.69**	2.00E-02
GO:0006412	Protein synthesis	0.27	1.00E-05	**3.39**	4.00E-18
GO:0006260	DNA synthesis/replication	0.25	n.s.	**2.74**	1.00E-02
GO:0005840	Ribosomal proteins	0.05	1.20E-07	**7.44**	1.00E-27

n.s., not significant; enrichment values >1.5 with a *P*-Value <0.05 are given in bold face.

The up- and down-regulated gene sets were then analyzed for enrichment for specific protein domains as annotated in the pfam database (Table 2). The up-regulated gene set was found to be enriched in the presence of three domains previously implicated in fungal incompatibility, the HET, the NACHT, and the HeLo domains. More than half of the 130 genes encoding HET domain proteins were found to be up-regulated. The HeLo domain is a toxicity domain found in the HET-S heterokaryon incompatibility protein (Greenwald *et al.* 2010) and the lopB pathogenicity protein of *Leptospheria maculans* (Idnurm and Howlett 2003). Also found are the lysM effector domain, a carbohydrate binding domain previously involved in host−parasite interactions in plants and fungi (Gruber *et al.* 2011; Gust *et al.* 2012; Martinez *et al.* 2012) and Hce2 effector domain (Stergiopoulos *et al.* 2012). *P. anserina* also displays six genes encoding proteins with a NPP1 domain, a pore-forming toxin functioning in pathogenicity in oomycetes and host defense in plants (Ottmann *et al.* 2009). Four of these genes are up-regulated. This list also contained DUF1996, a domain described as preferentially expressed during ectomycorrhiza formation (Martin *et al.* 2010). Also enriched are various domains involved in ion transport (Ctr, Ammonium_transp, Cation_ATPase). All six genes encoding the Ctr copper transporter domain are up-regulated. Pfam-A enrichment list also contains two domains with hydrolase activity, the Patatin phospholipase domain and an α/β hydrolase domain.

**Table 2:** List of Pfam-A domains enriched in the up-regulated gene set

Pfam Identification	Pfam Name	Functional Category	Number of Up-Regulated Genes With pfam-A	Total Number of Genes With pfam-A	Fraction of Up-Regulated Genes, %	P-Value
PF06985	HET	Incompatibility	68	130	52	7.95E-15
PF05729	NACHT	Incompatibility. Signal transduction	19	34	56	1.20E-05
PF12796	Ank_2	Protein−protein interaction	32	77	42	6.70E-05
PF04145	Ctr	Transport	6	6	100	9.75E-05
PF14479	HeLo	Pore-forming toxin	7	9	78	2.60E-04
PF01734	Patatin	Degradation	6	9	67	0.0045
PF00909	Ammonium_transp	Transport	5	7	71	0.0065
PF00550	PP-binding	Secondary metabolism	13	30	43	0.0066
PF00023	Ank	Protein−protein interaction	9	18	50	0.0069
PF09362	DUF1996	Unknown	12	27	44	0.0077
PF05199	GMC_oxred_C	Oxidoreduction	14	33	42	0.0088
PF00689	Cation_ATPase_C	Transport	6	10	60	0.0091
PF14856	Hce2	Pathogen effector	5	8	62	0.0095
PF13460	NAD_binding_10	Oxidoreduction	7	13	54	0.0105
PF00732	GMC_oxred_N	Oxidoreduction	13	31	42	0.0134
PF05630	NPP1	Necrosis inducer	4	6	66	0.0153
PF00690	Cation_ATPase_N	Transport	6	11	55	0.0165
PF13637	Ank_4	Protein−protein interaction	6	11	55	0.0165
PF01476	LysM	Defense	8	17	47	0.0167
PF08659	KR	Secondary metabolism	7	14	50	0.0171
PF02668	TauD	Oxidoreduction	10	23	43	0.0185
PF01565	FAD_binding_4	Oxidoreduction	15	40	38	0.0194
PF12697	Abhydrolase_6	Degradation	15	40	38	0.0194

Several domains involved in oxido-reduction processes (GMC_oxred, NAD_binding _10, FAD_binding_4 and TauD) as well as domains involved in polyketide synthase-based secondary metabolism (KR, PP-binding) are all enriched. This observation prompted us to analyze expression of secondary metabolite clusters. We have compiled the genes belonging to secondary metabolite synthesis clusters and found that in that set of 185 genes (belonging to 35 clusters), 63 were up-regulated indicating a significant enrichment for genes belonging to such clusters (*P* = 4.10^−7^). Table S2 gives a list of the secondary metabolism clusters up-regulated during incompatibility. Also up-regulated was the ortholog of the *Lae1* secondary metabolism regulator characterized in several fungal species (Butchko *et al.* 2012).

On the basis of the enrichment of the NACHT domain and the implication of STAND proteins in various incompatibility systems in *P. anserina* and also *C. parasitica* (Paoletti *et al.* 2007; Chevanne *et al.* 2009; Choi *et al.* 2012), we compiled the ensemble of *P. anserina* genes encoding NACHT or NB-ARC domains. A total of 62 such genes were identified and 32 of those were found to be up-regulated during incompatibility. Table 3 lists a subset of these NACHT and NB-ARC domain proteins with a typical STAND protein organization. Five of these STAND proteins correspond to the previously identified NWD proteins (NWD1, NWD2, NWD5, HNWD1, and HET-D), which share strongly conserved WD-repeats that evolve by concerted evolution and under a positive selection regimen (Paoletti *et al.* 2007). A large proportion of the proteins include N-terminal domains with a predicted enzymatic function, five genes encode proteins with an N-terminal putative lipase domain related to *sesB* of *Nectria hematococca* (Graziani *et al.* 2004), one protein shows a patatin phospholipase domain, and two proteins show a purine nucleoside phosphorylase domain. The NACHT domains are associated with various types of super-structure forming repeat domains (WD, ANK, and HEAT repeats). A single STAND protein in this list has a NB-ARC domain instead of a NACHT domain and this domain is found associated to TPR repeats. The NB-ARC/TPR association has been observed before in fungal STAND proteins (Martin *et al.* 2010; Daskalov *et al.* 2012). The *het-R/het-V* incompatibility reaction, which involves a STAND protein, leads to the induction of a large and various set of other STAND proteins, including several proteins involved in other incompatibility systems (*het-D* and *nwd2*). Thus STAND proteins appear to be both upstream triggers of the incompatibility reaction and downstream-induced factors, a characteristic they share with HET domain proteins.

**Table 3:** List of up-regulated STAND proteins

Gene	Alternate Designation	Max. Fold Change	Size (aa)	N-Terminal Effector Domain	Central NOD Domain	C-Terminal Repeat Domain
Pa_5_8000		93.5	1060	Unknown	NACHT	None detected
Pa_5_7830		17.74	1138	Unknown	NACHT	None detected
Pa_2_8180		4.94	1179	PNP_UDP_1	NACHT	ANK
Pa_1_11380		4.65	1384	Patatin	NB-ARC	TPR
Pa_6_7270		3.97	1422	sesB-like	NACHT	HEAT
Pa_5_12100		3.88	1562	sesB-like	NACHT	WD
Pa_7_5020		3.78	1046	Unknown	NACHT	None detected
Pa_4_1190	*hnwd1*	3.64	1511	HET	NACHT	WD
Pa_5_1230	*nwd5*	3.62	1074	RelA_SpoT	NACHT	WD
Pa_2_7940	*het-d*	3.52	1124	HET	NACHT	WD
Pa_7_10370		3.06	1404	sesB-like	NACHT	ANK
Pa_3_9600		3.02	1712	sesB-like	NACHT	WD
Pa_3_8560		2.94	1207	PNP_UDP_1	NACHT	ANK
Pa_3_10930	*nwd1*	2.34	1314	NAD1	NACHT	WD
Pa_3_640	*nwd2*	2.26	1118	HET-s PFD-like	NACHT	WD
Pa_0_150		2.07	2018	Unknown	NACHT	ANK
Pa_5_11700		2.04	1041	sesB-like	NACHT	None detected

Table S3 lists the 100 genes with the greatest fold changes (ranging from 401 to 38). Based on this list, there appears to be a strong up-regulation of various hydrolase activities. The list includes in particular six proteases and four lipases. The list also includes four phenoloxidases, which is in agreement with previous biochemical studies in which authors reported up-regulation of proteases and phenoloxidases activities (including laccases) during incompatibility (Bégueret and Bernet 1973; Boucherie and Bernet 1977).

In the down-regulated gene set, consistent with the results of the GO term enrichment analysis (Table 1), enriched pfam-A domains corresponded to domains involved in ribosome biogenesis (MMR_HSR1, DEAD, Ribosomal_L7Ae, Brix, Helicase_C), DNA replication (MCM), and mitochondrial function (Mito_carr); Table S4. The list of genes encoding carbohydrate active enzymes was recovered from the CAZy database and analyzed for gene expression changes (Table S5). Globally, in the different categories more down-regulated than up-regulated enzymes were found. Exceptions to that trend were subcategories CE3, GH16, GH18, GH76, GT1, GT2, GT31, CBM18, and CBM50 (lysM) for which up-regulated genes were more frequent than down regulated genes.

Globally, the functional annotation of the up-regulated gene set, indicates an up-regulation of hydrolytic activities in particular lipases and proteases, an up-regulation of a set of STAND signal transduction proteins and numerous HET domain proteins as well as an induction of several secondary metabolism clusters. Of note is also the enrichment for lysM domain proteins. The down-regulated gene set indicates a down regulation of mitochondrial function, translation and ribosome synthesis.

### Up-regulation of toxin and effector-like proteins

Fungal genomes encode large sets of variable cysteine-rich extracellular proteins several of which have effector or toxin activities. Because the first *idi* genes identified in a subtractive hybridization approach, *idi-1*, *idi-2*, and *idi-3*, encode small extracellular proteins (Bourges *et al.* 1998; Dementhon *et al.* 2003), we analyzed the enrichment in the up-regulated gene set for small extracellular proteins (<250 aa) (Table 4). We found a 1.61-fold enrichment for small extracellular proteins in the up-regulated gene set (*P* = 0.001). The enrichment reached 2.2-fold when small cysteine-rich proteins (cysteine content >6%) were considered.

**Table 4:** Enrichment for small extracellular cysteine-rich proteins in the up-regulated set

	Up-Regulated/Total	% Up-Regulated	Enrichment In Up-Regulated Set	*P*-Value
Extracellular	190/781	24.3	1.15	0.07
Small extracellular (<250 aa)	85/250	34.0	1.61	0.001
Small extracellular (<250 aa) and cys-rich (>3.5%)	42/103	40.8	1.93	0.002
Small extracellular (<250 aa) and cys-rich (>6%)	19/40	47.5	2.24	0.008

The *idi-2* gene encodes a protein homologous to victoriocin an antifungal toxin produced by *Helminthosporium victoriae* (de Sa *et al.* 2010a,b). In addition, the pfam functional annotation of the up-regulated genes, identified an enrichment for the HeLo domain, the toxicity domain of the HET-S protein, which behaves as a pore-forming toxin (Greenwald *et al.* 2010; Mathur *et al.* 2012; Seuring *et al.* 2012) and is also found in the LopB pathogenicity protein of *L. maculans* (Idnurm and Howlett 2003), the *Hce2* effector domain (Stergiopoulos *et al.* 2012) and the NPP1 toxin domain (Ottmann *et al.* 2009). This prompted us to analyze the set of up-regulated genes for presence of other genes with a potential toxin or effector-like activity. Table 5 gives a list of such genes identified in the up-regulated gene set. In addition to HET-S, there are eight additional proteins with a HeLo domain, of which seven are up-regulated during incompatibility. HeLo domain proteins can thus be involved in the triggering of the incompatibility reaction (in the *het-s/het-S* system) but also correspond to downstream transcriptionally activated target genes. HET-S is not up-regulated, but NWD2, its proposed functional partner, is (Table 3) (Daskalov *et al.* 2012). Then, a gene encoding a protein with a aerolysin-like pore-forming domain is up-regulated 18-fold. This protein also displays a lysM domain and a jacalin-like lectin domain. Homologs of the PR-1 and cerato-platanin effector proteins described in plant pathogens are up-regulated (Pazzagli *et al.* 1999) and two ion channel inhibitor toxins, a small peptide of 30 amino acids in length (Pa_6_9450), related to the Ptu family, and a gene related to the KP4 toxin family are also up-regulated (Bernard *et al.* 2001; Brown 2011) .

**Table 5:** Induced genes and gene families encoding toxins or effector-like proteins

Domain	Activity	Genes	Size (aa)	Max. Fold Change	Associated Domains	Signal Peptide
Victoriocin-like	Antifungal toxin					
		idi-2	157	**3.97**		Yes
HeLo	Pore-forming toxin					
		Pa_2_4880	664	**isp**		No
		Pa_7_900	449	**14.02**	Rho	No
		Pa_5_9905	165	**8.94**		No
		Pa_4_8870	652	**6.55**	Pkc-like	No
		Pa_5_11500	354	**6.15**	P-loop	No
		Pa_6_8690	630	**4.31**	Pkc-like	No
		Pa_7_4390	1236	**2.58**		No
		het-S	289	1.18	het-s PFD	No
		Pa_7-7510	639	0.42	Pkc-like	No
NPP1	Pore-forming toxin					
		Pa_2_8010	243	**34.16**		Yes
		Pa_3_10530	276	**31.79**		Yes
		Pa_5_1900	302	**8.66**		Unclear
		Pa_5_1110	285	**5.89**		Yes
		Pa_1_15230	168	1.18		Yes
		Pa_5_10750	271	0.43		No
Aerolysin-like	Pore-forming toxin					
		Pa_5_2020	447	**17.85**	Jacalin-like lectin and lysM	Yes
PR-1	Effector					
		Pa_4_2780	198	**12.66**		No
Cerato-platanin	Effector toxin					
		Pa_2_4030	138	**3.83**		Yes
Hce2	Putative effector					
		Pa_6_10940	204	**9.81**		Yes
		Pa_6_10945	185	**6.81**		Yes
		Pa_5_10540	172	**3.32**		Yes
		Pa_5_1150	1350	**3.07**	Chitin binding and GH18	Yes
		Pa_3_10455	206	**2.19**		Yes
		Pa_2_4765	184	1.73		Yes
		Pa_2_4722	207	1.38		Yes
		Pa_1_17065	131	1.23		Yes
Kp4 killer toxin	Calcium channel inhibitor					
		Pa_5_3912	166	**42.76**		Yes
Ptu assassin bug toxin	Ion channel inhibitor					
		Pa_6_9450	30	**39.85**		No

Fold change values >2 are given in bold face.

### Comparison with *N. crassa*

**Figure 4:**
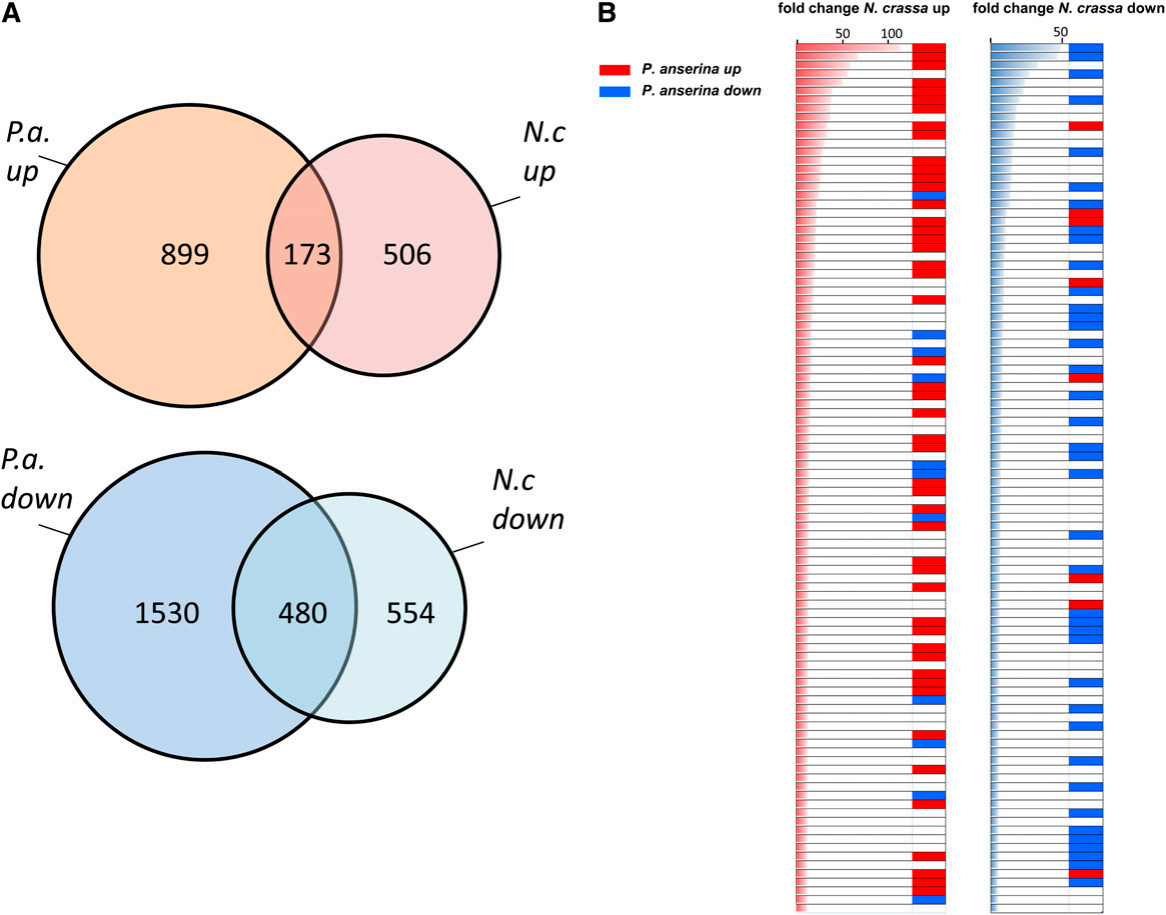
Overlap in the transcriptional response to incompatibility in *P. anserina* and *N. crassa*. (A) Venn diagrams of the overlap between the transcriptional responses in *P. anserina* and *N. crassa* for the set of orthologous gene pairs are given for the up and down regulated genes respectively in pink and blue. (B) 100 *N. crassa* genes showing a *P. anserina* ortholog and the highest fold change values in the Hutchison *et al.* (2009) study were ordered by fold change value. For each diagram, the bar on the left represents the value of the fold change, the up and down regulated group is given in red and blue, respectively. For each gene, the expression status of the corresponding *P. anserina* homolog is given on the right as a red bar for an up-regulated gene and a blue bar for a down-regulated gene or a blank for FC values <2.

Next, we compared the transcriptional response to incompatibility in *P. anserina* with the results of a previous study in *N. crassa* (Hutchison *et al.* 2009). This comparison was made for the fraction of genes that are common to both genomes and form orthologous gene pairs. Among the 10556 *P. anserina* CDS analyzed on microarrays, a total of 6873 had an ortholog in *N. crassa*. The majority of the genes up-regulated in *P. anserina* were not up-regulated in *N. crassa* (Figure 4A). Yet, we found a significant correlation (*P* < 10^−4^) between the gene set up-regulated during incompatibility in *N. crassa* and *P. anserina*. A total of 173 orthologous gene pairs were up-regulated both in *P. anserina* and *N. crassa* (Figure 4A and Table S6). The overlap was more pronounced in the down-regulated gene sets, with 480 orthologous pairs down regulated in both species (Figure 4A; *P* < 10^−10^). These observations indicate that the transcriptional responses in both species are not identical but nevertheless related. The correlation between the gene up-regulation during incompatibility in *P. anserina* and *N. crassa* became more evident when genes with the highest fold change were considered. For instance, among the 100 up-regulated genes showing the highest fold change in *N. crassa*, 48 were also up-regulated in *P. anserina* (Figure 4B). In the list of 173 genes up-regulated in both species (Table S6), one finds four genes involved in autophagy suggesting that induction of autophagy might be common to both systems. Other interesting genes in this list are the Zn finger transcription regulators belonging to expression cluster H mentioned above which are up-regulated early and transiently in *P. anserina* (Pa_1_18880/NCU00694 and Pa_3_1720/ NCU07952) and the STAND protein with a patatin domain (Pa_1_11380/NCU09244) especially because this gene was described as a heterokaryon incompatibility gene in *C. parasitica* (Choi *et al.* 2012). Then, two genes SPA1 (Pa_1_14890) and SPA17 (Pa_6_4800) belonging to the recently defined SPA-family (for septal pore-associated) involved in septal plugging in *N. crassa* are found (Lai *et al.* 2012). SPA1 was shown to aggregate and localize to the septal pore during incompatibility in *N. crassa* (Lai *et al.* 2012). In addition to the SPA1 and SPA17 orthologs, orthologs of SPA4, 11, 14, and 15 are also up-regulated during incompatibility in *P. anserina*. In the list of 480 common down regulated genes, one finds virtually all genes encoding ribosomal proteins, a large number of genes involved in mitochondrial respiration and primary metabolism (Table S6). It appears that a general shutdown of ribosome synthesis and mitochondrial function occurs in both systems. We have analyzed pfam-A term enrichment and GO term enrichment in the common up and down regulated gene set (Table S7 and Table S8). The up-regulated genes common to both species were found enriched for pfam-A domains related to ubiquitin-based protein degradation. In addition, there was an enrichment for adenosine deaminases (*P* = 4.10^−5^). Enriched GO terms were “protein modification” (*P* = 0.005) and “proteolysis and peptidolysis” (*P* = 0.01). In the down-regulated gene set, many pfam-A domains associated with ribosomal proteins were enriched. Enriched GO terms were “carbohydrate metabolism” (*P* = 0.0002), “protein synthesis” (*P* = 0.007), “ribosomal proteins” (*P* = 5.10^−9^), and “energy/TCA cycle” (*P* = 2.10^−10^).

Globally, although the transcriptional responses differ in both systems a number of common regulated genes can be identified. In both systems, incompatibility appears to be related with up-regulation of protein degradation processes. The down-regulation transcriptional responses show a clear overlap in both species and involve down regulation of mitochondrial function and ribosome synthesis.

### Up-regulated genes preferentially locate on specific chromosomes and in chromosomal islands

By visual inspection of the up-regulated gene lists, we noticed many situations in which up-regulated genes are organized as strings of adjacent genes. We have thus analyzed the genomic distribution of the up-regulated genes. We compared the observed occurrence of such expression gene clusters to the expected occurrence of clusters of adjacent up-regulated genes in random distribution. Clusters of three or more adjacent up-regulated genes occurred more frequently than expected at random (Figure S1 and Table S9). This bias increases with the size of the clusters. A total of 15 clusters of 7 genes or more are found (Table S10). Only a fraction (2/15) of these large up-regulated gene clusters corresponded to putative secondary metabolite gene clusters while others are distinct and bear no apparent relation with secondary metabolism clusters. The largest cluster comprises 10 adjacent up-regulated genes. The down-regulated genes showed a much lower tendency to genomic clustering. Only one cluster of six genes or more was present. In total, 16% of the up-regulated genes are found in clusters of four or more up-regulated genes (3% expected in a random distribution), this proportion was 6.6% in the down-regulated set (4% expected in a random distribution).

Next we have analyzed the distribution of up- and down-regulated genes by chromosome and found a significant enrichment of up-regulated genes on chromosome IV and V (Table 6). The same chromosomes were also found to be enriched for extracellular proteins, genes lacking orthologs in *N. crassa*, genes encoding HET domain proteins, and transposon sequences. Conversely, on chromosome I and II, the same categories were found to be underrepresented. Chromosome IV and V are also the chromosome containing the majority of the secondary metabolism clusters. Chromosome V is also enriched for NACHT domain encoding genes (*P* < 0.05) and *isp* genes (*P* < 10^−3^). There thus appears to exist a chromosome specialization in relation to the incompatibility response in *P. anserina*, with in particular chromosome V being enriched in genes up-regulated during incompatibility and genes encoding HET domains and NACHT domains.

**Table 6:** Chromosome-specific enrichment in up-regulated genes on chromosomes IV and V

Chr.	Size, Mb	Number of Genes	Number of Up-Regulated Genes	% of Up-Regulated Genes	Enrichment in Up-Regulated Genes	*P*-Value	Number of Genes Encoding Extracellular Proteins	Enrichment for Genes Encoding Extracellular Proteins	*P*-Value	Number of Genes w/o Ortholog In N. C.	Enrichment in Genes w/o Ortholog In N. C.	*P*-Value	Number of Genes Encoding HET Domains	Enrichment in Genes Encoding HET Domains	*P*-Value	Number of Transposons	Transposons per Mb	Enrichment in Transposons	*P*-Value	Number of Secondary Metabolism Clusters
I	8.8	2557	533	20.84	0.97	0.0039	146	0.76	6E-05	706	0.76	6E-28	15	0.47	3E-04	109	12.37	0.64	3E-05	4
II	5.1	1569	281	17.91	0.83	2E-04	118	1.00	n.s.	475	0.83	2E-08	15	0.76	n.s.	59	11.42	0.59	5E-04	5
III	4.7	1208	230	19.04	0.89	0.0306	83	0.91	n.s.	428	0.97	n.s.	16	1.06	n.s.	82	17.40	0.90	n.s.	3
IV	3.8	1119	293	26.18	**1.22**	7E-05	111	**1.33**	0.0014	488	**1.19**	3E-07	20	1.43	0.08	106	27.84	**1.43**	1E-03	8
V	4.7	1447	372	25.71	**1.20**	3E-05	167	**1.54**	3.7E-09	769	**1.45**	1E-43	37	**2.04**	2E-05	204	43.09	**2.22**	2E-13	11
VI	4.2	1277	280	21.93	1.02	n.s.	68	0.71	0.0012	539	1.16	8E-6	19	1.19	n.s.	87	20.40	1.05	n.s.	3
VII	4.1	1217	242	19.88	0.93	n.s.	88	0.96	n.s.	393	0.88	0.0011	8	0.53	0.05	45	11.01	0.57	1E-03	1

n.s., not significant.

Enrichment values on Chr IV and V with a significant *P*-Value <0.05 are given in bold.

**Figure 5:**
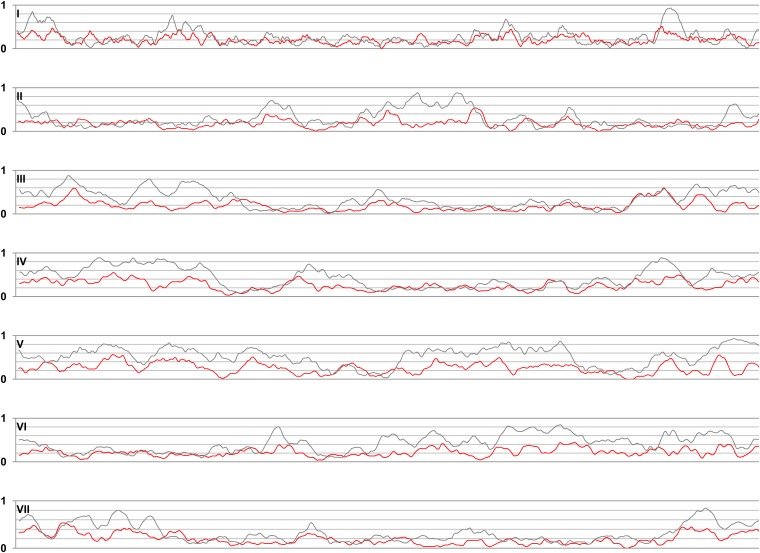
Chromosomal distribution of up-regulated genes and of genes lacking orthologs in *N. crassa*. Each of the seven *P. anserina* chromosomes was analyzed in a sliding-window analysis for the density of up-regulated genes (red line) and for the density of genes lacking homologs in *N. crassa* (gray line). Window size is 30 genes. Density is expressed as the fraction of up-regulated gene (or fraction of genes lacking an ortholog in *N. crassa*) in each window. The horizontal axis gives the position of the genes along the chromosome, the vertical axis, the fraction of up-regulated genes or the fraction of genes without ortholog in each 30 gene sliding window.

To further explore this biased territorial distribution of up-regulated genes, we have analyzed distribution of up-regulated genes along the chromosomes (Figure 5). Distribution of up-regulated genes was found to be heterogeneous on all chromosomes and characterized by the presence of large genomic regions enriched in up-regulated genes. There was a partial correlation between regions enriched in genes showing no ortholog in *N. crassa* and those enriched for up-regulated genes. On some chromosomes like chromosome VII, there was a trend of greater density of up-regulated (and poorly conserved genes) toward the subtelomeric areas but intrachromosomal islands rich in up-regulated genes are also found for instance on chromosomes IV and V. Together, these analyses indicate that genes up-regulated during incompatibility tend to cluster onto specific chromosomes and genomic regions.

## Discussion

Herein, we have analyzed, on a genome-wide scale, the transcriptional changes associated with recognition of nonself as occurring during the heterokaryon incompatibility response in the fungus *P. anserina*. We find that more than 21% of the genes undergo an up-regulation by a fold of 2 or more whereas a similar proportion of the genome is down-regulated. Some level of similarity in the responses is apparent when *P. anserina* and *N. crassa* are compared (Hutchison *et al.* 2009). The analysis of the up-regulated gene set increases our global understanding of the incompatibility response in fungi. The response is complex and involves many genes showing poor evolutionary conservation and residing preferentially on specific chromosomes or chromosome regions. The ensemble of up-regulated genes bears notable similarities with genes that have been implicated in interspecific biotic interactions (pathogenicity, mutualism, mycoparasitism, competition, or host-defense) in other fungal species.

### Similarities and differences between the incompatibility responses in *N. crassa* and *P. anserina*

Heterokaryon incompatibility appears near ubiquitous in filamentous fungi. Incompatibility reactions have been identified essentially in all species that were tested for this activity. This conservation brings about the question of the mechanistic similarity of the incompatibility reaction in different species. Cytologically, vacuolization, and compartmentation have been described as common traits of the incompatibility reaction in various species (Glass and Kaneko 2003; Pinan-Lucarre *et al.* 2007). In addition, as already mentioned there is a common involvement of HET domain and STAND proteins in incompatibility in several species (Aanen *et al.* 2010; Choi *et al.* 2012).

The transcriptional responses in incompatibility in *N. crassa* and *P. anserina* are comparable in terms of number of genes involved with roughly 1000−2000 genes up- or down-regulated in each species (Hutchison *et al.* 2009). Gene expression changes associated with programmed cell death inducing drugs staurosporine and phytosphingosine have also been analyzed in *N. crassa* (Videira *et al.* 2009; Fernandes *et al.* 2011). The transcription changes to these PCD inducing drugs differed from the transcriptome changes induced by incompatibility in *N. crassa* and *P. anserina*. In particular, the numbers of induced and repressed genes were much lower than observed in incompatibility, with for instance a total of only 135 genes showing gene expression changes in the presence of staurosporine as compared to several thousand in the incompatibility situation.

The transcriptional responses to incompatibility are different but yet related in the two species. The transcriptional responses differ in the two species for the simple reason that the majority of the genes that are up-regulated in *P. anserina* have no unambiguous ortholog in *N. crassa*. Yet 173 orthologous pairs were found to be up-regulated in both species. When considering the genes with the highest fold change in *N. crassa*, the correlation markedly increases. Induction of autophagy during incompatibility has been showed by cytologic approaches in *P. anserina* (Pinan-Lucarre *et al.* 2003). There is as yet no direct evidence for the induction of autophagy during incompatibility in *N. crassa*, but the fact that several autophagy genes are up-regulated during incompatibility both in *N. crassa* and *P. anserina* suggests that induction of autophagy could be a common manifestation of incompatibility. Gene families, protein domains and gene ontology terms enriched in both species should be considered with special interest since these may reflect conserved core components of the incompatibility response. In that sense, induction of proteolytic and lipolytic activities appears common in both systems. For instance, the patatin-like phospholipase STAND protein (Pa_1_11380/NCU09244) is common to both systems. Also of interest are two Zn-finger transcription factors (Pa_1_18880/NCU00694 and Pa_3_1720/ NCU07952) showing an early and transient induction in *P. anserina*. These genes may constitute conserved upstream regulators of the transcriptional response. Also common to both species is the up-regulation of adenosine deaminases enzymes.

When the down-regulated gene sets are considered the overlap in the two species is more pronounced. This can in part be attributed to the fact that down regulated gene set in *P. anserina* is enriched for genes showing orthologs in *N. crassa* and other fungi. In both species, there appears to be a down-regulation of genes involved in ribosome biogenesis, protein synthesis, and energy production, suggesting that in both species the incompatibility reaction corresponds to a global halt of core cellular maintenance activities while resources are re-allowed to novel activities that are more species specific.

### Fungal STAND proteins in incompatibility and beyond

The incompatibility reaction described in the present paper is controlled by the interaction of the *het-R* and *het-V* incompatibility genes. Het-R encodes a STAND protein of the *nwd* gene family and displays an N-terminal HET domain (Chevanne *et al.* 2009). Several of the known incompatibility systems in *N. crassa*, *P. anserina*, and *C. parasitica* involve proteins encoding HET domains (Saupe *et al.* 1995; Shiu and Glass 1999; Espagne *et al.* 2002; Kaneko *et al.* 2006; Chevanne *et al.* 2009; Choi *et al.* 2012). This domain shows a considerable expansion in the genome of many filamentous fungi (Fedorova *et al.* 2005). Although the HET domain was originally identified in proteins encoded by fungal heterokaryon incompatibility genes, the number of HET proteins in fungal genomes in general greatly exceeds the number of genetically identified *het* loci. This raises the question of the function of this plethora of HET domain proteins. The *P. anserina* genome encodes 130 HET domain proteins. We find that >52% of these genes are up-regulated during incompatibility. The same remark applies to the STAND proteins. In this system, a STAND protein (HET-R) is the upstream inducer of the incompatibility response, which in turn induces transcriptional activation of a large set of other STAND proteins including another known *het* gene, namely *het-d*. This principle is illustrated by the Pa_1_11380 gene, which encodes a STAND protein with patatin-like phospholipase effector domain. This STAND protein is up-regulated during incompatibility both in *P. anserina* and *N. crassa* and was found to control *vic2* incompatibility in *C. parasitica* (Hutchison *et al.* 2009; Choi *et al.* 2012). It appears that gene encoding STAND proteins (and HET and HeLo domain proteins) can be involved in incompatibility both as upstream triggers and as downstream transcriptionally-activated target genes.

The particular status of the fungal STAND proteins is underscored by the fact that in other species, in addition to *P. anserina*, these genes have been shown to be polymorphic and rapidly evolving and subject to extensive expansion in paralogous gene families (Fedorova *et al.* 2008; Martin *et al.* 2008; Burmester *et al.* 2011; Kubicek *et al.* 2011; Zuccaro *et al.* 2011; Iotti *et al.* 2012). STAND proteins show a marked gene expansion in the endophyte *Pirinoformospora indica* (Zuccaro *et al.* 2011) and mycoparasitic trichoderma species (Kubicek *et al.* 2011) but also in ectomycorrhizal species like *L. bicolor* and *T. melanosporum*. In these species, a large set of these STAND proteins (of the NB-ARC TPR type) are up-regulated in ECM (Martin *et al.* 2010). In *Tuber melanosporum*, an expanded *nank* (NACHT ANK) family is, in addition, characterized by a remarkable diversification mechanism based on alternative splicing of tens of codon-sized microexons (Iotti *et al.* 2012). Fungal STAND proteins have been proposed to serve as receptors for heterospecific nonself cues (Paoletti *et al.* 2007; Paoletti and Saupe 2009), this proposed function might explain both their high polymorphism and rapid diversification and their expansion in certain species critically dependent on biotic interactions. Although fungal STAND proteins have been initially identified in the context of heterokaryon incompatibility (Saupe *et al.* 1995), it now seems reasonable to envision that their general function in fungi relates to various forms of recognition of nonself in a variety of interorganismal interactions, both between individuals of the same species and of different species.

### Incompatibility resembles a response to heterospecific nonself

The incompatibility response is characterized by the induction of proteolytic, lipolytic, and phenoloxidases activities. In addition to these activities a set of toxins, effector-like small cysteine-rich proteins and secondary metabolism clusters are up-regulated in incompatibility. Enriched pfam domains include the NACHT, the HET, and the HeLo, lysM, Hce2, and NPP-1 domains. Up-regulated genes show a clustered chromosomal distribution and overall low evolutionary conservation. Collectively, these sequence signatures and functional features have been previously found to be associated with genes involved in pathogenicity, mutualism or niche competition in other fungal species (Fox and Howlett 2008; Berendsen *et al.* 2010; Martin *et al.* 2010; de Jonge *et al.* 2011; Schmidt and Panstruga 2011; Veneault-Fourrey and Martin 2011). Secondary metabolism clusters, species-specific genes, small secreted cysteine-rich proteins acting as effectors, toxins, proteases, lipases, and phenoloxidases have been associated with biotic interactions such mutualism, pathogenicity and host-defense. More specifically, in mycoparasitic Trichoderma species there is a gene expansion for numerous domains that are found to be enriched in the up-regulated gene set including HET and NACHT domain proteins, ANK repeat proteins, lysM domain effector proteins (Kubicek *et al.* 2011). Similarly, in the endophyte *Piriformospora indica* there is a considerable expansion of NWD proteins with 99 ORFs encoding such STAND proteins (Zuccaro *et al.* 2011). As already mentioned in the ectomycorhizal species like *L. bicolor* and *T. melanosporum* there is also an expansion and a transcriptional induction of STAND proteins (Martin *et al.* 2010). A domain of unknown function (DUF1996) was found to be enriched both during ECM formation (Martin *et al.* 2010) and incompatibility in *P. anserina*. Of note is also the fact that genes induced during incompatibility reside preferentially on specific chromosomes and within chromosomal islands a situation that has now been repeatedly reported for genes related to pathogenicity in other fungal species (Fedorova *et al.* 2008; Ma *et al.* 2010; Rep and Kistler 2010; Raffaele and Kamoun 2012).

Typically incompatibility is understood as a mechanism aimed at the destruction of the cell resulting from the fusion between incompatible individuals from the same species. In this context the magnitude and complexity of the transcriptional response during incompatibility strikes as over-dimensioned and so to say excessive. How is this induction of a pleiotropic response (involving secondary metabolites, putative toxins and extracellular effectors and a large spectrum of lytic activities) to be understood if the role of incompatibility is simply to prevent that limited cell fusion event? We have proposed, essentially on the basiss of the homology existing between *het*-gene−encoded STAND proteins and pathogen recognition receptors of the animal NLR or plant NBS-LRR type, that the incompatibility machinery might have its original function in the detection and response to heterospecific nonself (Paoletti and Saupe 2009). The evolutionary ancestry between allorecognition and xenorecognition remains a general and debated question (Rinkevich 2004; Aanen *et al.* 2008). Pathogen-driven selection on these STAND proteins might represent an external selective force, allowing for the maintenance of polymorphism in these allorecognition genes that is otherwise problematic as originally noted by Crozier and others (Crozier 1986; Rousset and Roze 2007; Aanen *et al.* 2008). Our interpretation of the pleiotropic transcriptional response occurring during incompatibility is that the response to conspecific nonself as occurring in incompatibility activates a cascade whose function could also lie in the response to heterospecific nonself (xenorecognition), be it in the form of mycoparasitism, endophytic lifestyle, competition or host-defense against a microbial pathogen. This cascade involves effector-like proteins, toxins, proteases, lipases, phenoloxidases, secondary metabolism clusters, all of which have been involved in different forms of interspecific interactions in other fungal species. Although *P. anserina* is essentially considered a coprophillic saprophyte it remains possible that this species is also a cryptic endophyte or capable of mycoparasitic activity. There have been reports describing *P. anserina* as an endophyte (Matasyoh *et al.* 2011). In addition, like other ascomycetes *P. anserina* could be the prey of mycophagic bacteria or fungal mycoparasites. Further studies should now be engaged at the inter-organismal level in order to explore the function of this large set of rapidly diverging genes induced here in the allorecognition context.

## Supplementary Material

Supporting Information
