# Supplementary material for: The Transcriptional Response to Nonself in the Fungus Podospora anserina
Source: G3 (Bethesda). 2013 Jun 1;3(6):1015–30. doi: 10.1534/g3.113.006262 (PMC3689799; doi:10.1534/g3.113.006262)
Supplement: Supporting Information [file supp_3_6_1015__index.html]

The Transcriptional Response to Nonself in the Fungus Podospora anserina — Supporting Information 

# The Transcriptional Response to Nonself in the Fungus *Podospora anserina*

## Supporting Information for Bidard, Clave, and Saupe, 2013

**Files in this Data Supplement:**

- Figure S1 - Clustering of upregulated genes (PDF, 77 KB)
- Table S1 - Expression data and additional information for all analysed CDS ordered by chromosome (.xls, 2.8 MB)
- Table S2 - Lists 9 clusters identified using antismash and up-regulated during the incompatibility reaction (.xls, 34 KB)
- Table S3 - List of the 100 genes with highest upregulation fold change (.xls, 45 KB)
- Table S4 - Lists the Pfam-A domains enriched in the regulated gene set, the number of genes bearing each domain, the fraction upregulated and the *p*-value of the enrichment (.xls, 33 KB)
- Table S5 - Lists the carbohydrate active enzyme (CAZy) families and gives the number of up- and down regulated genes and the total number of genes in each family (.xls, 30 KB)
- Table S6 - Lists of orthologous gene pairs that are up (or down) regulated during incompatibility in *P. anserina* and *N. crassa* (.xls, 138 KB)
- Table S7 - Lists the enriched Pfam-A domains, the number of genes bearing each domain, the fraction of up- or down regulated genes and the *p*-value of the enrichment in up and down regulated genes in *P. anserina* and *N. crassa* incompatibility (.xls, 36 KB)
- Table S8 - Enriched GO terms in the set of orthologous gene pairs up or down regulated in *N. crassa* and *P. anserina* with enrichment p-value ≤0.01 (.xls, 30 KB)
- Table S9 - Number of clusters of adjacent up and down regulated genes observed experimentally and expected in a random distribution (.xls, 34 KB)
- Table S10 - Lists the 15 clusters of 7 or more adjacent upregulated genes (.xls, 38 KB)
