# Supplementary material for: The Transcriptional Response to Nonself in the Fungus Podospora anserina
Source: G3 (Bethesda). 2013 Jun 1;3(6):1015–30. doi: 10.1534/g3.113.006262 (PMC3689799; doi:10.1534/g3.113.006262)
Supplement: Supporting Information [file supp_g3.113.006262_FigureS1.pdf]

A

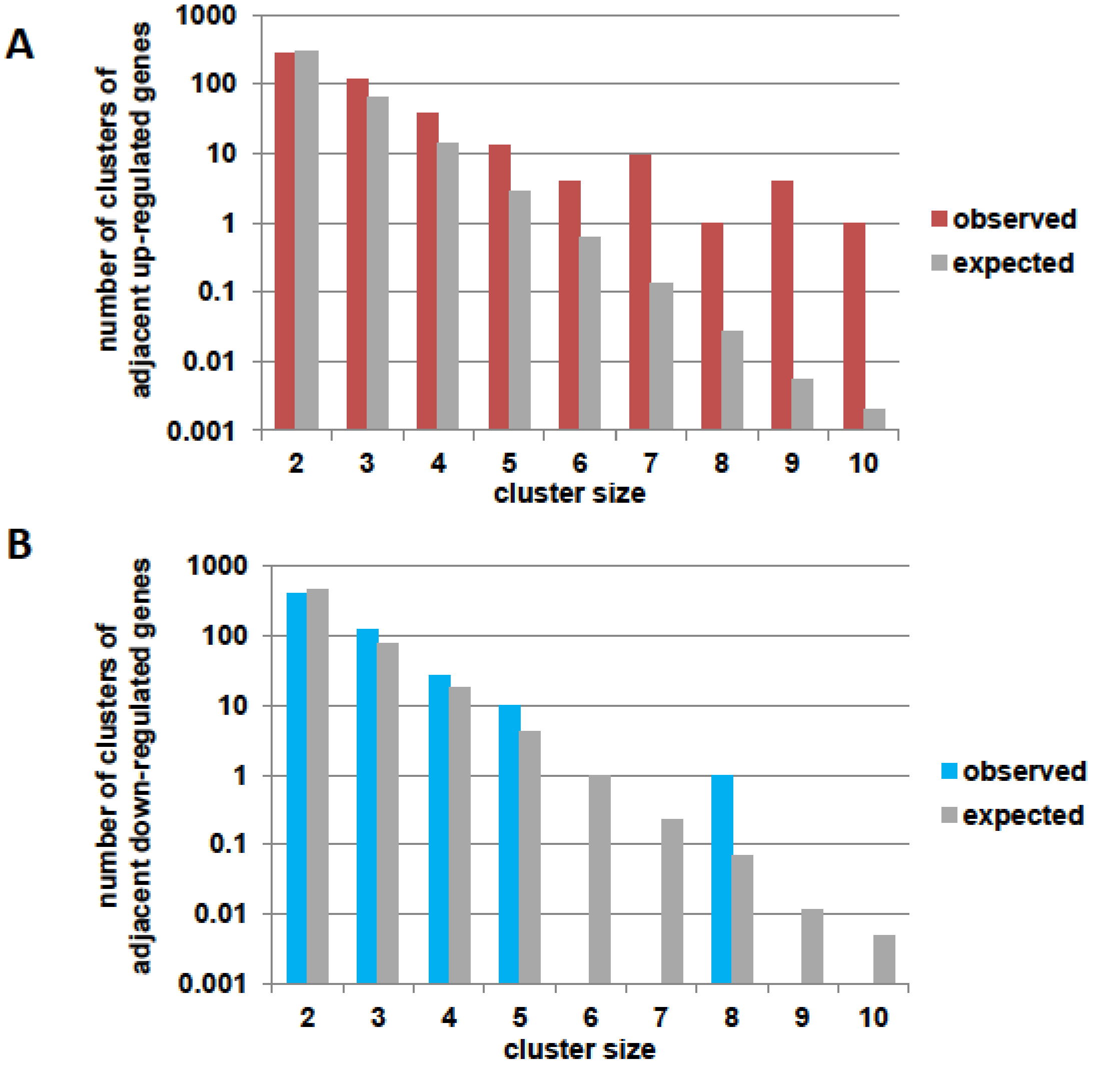

Figure S1 Clustering of upregulated genes. In A, the expected and observed number of clusters of 2 to 10 adjacent upregulated genes are given. In B, the expected and observed number of clusters of 2 to 10 adjacent down regulated genes are given.
